# Evidence of Lifestyle Modification in the Management of Hypercholesterolemia

**DOI:** 10.2174/157340313805076313

**Published:** 2013-02

**Authors:** GS Mannu, MJS Zaman, A Gupta, Rehman HU, PK Myint

**Affiliations:** 1 Norfolk and Norwich University Hospital, Norfolk, U.K; 2 James Paget University Hospital, Great Yarmouth, Norfolk, U.K; 3 The George Institute for Global Health, Sydney, Australia; 4 Department of Medicine, West Wales Hospital, Carmarthen, Wales, U.K; 5 Regina Qu"Appelle Health Region, Regina, SK, Canada; 6 Norwich Research Park Cardiovascular Group, Norwich Medical School, University of East Anglia, Norwich, U.K

**Keywords:** Coronary heart disease (CHD), hypercholesterolemia, CVD risk, lifestyle modification.

## Abstract

**Background:**

Coronary heart disease (CHD) is the leading cause of morbidity and mortality worldwide. The growth of ageing populations in developing countries with progressively urbanized lifestyles are major contributors. The key risk factors for CHD such as hypercholesterolemia, diabetes mellitus, and obesity are likely to increase in the future. These risk factors are modifiable through lifestyle.

**Objectives:**

To review current literature on the potential benefit of cholesterol lowering in CHD risk reduction with a particular focus on the evidence of non-pharmacological/lifestyle management of hypercholesterolemia.

**Methods:**

Medline/PubMed systematic search was conducted using a two-tier approach limited to all recent English language papers. Primary search was conducted using key words and phrases and all abstracts were subsequently screened and relevant papers were selected. The next tier of searching was conducted by (1) reviewing the citation lists of the selected papers and (2) by using PubMed weblink for related papers. Over 3600 reports were reviewed.

**Results:**

Target cholesterol levels set out in various guidelines could be achieved by lifestyle changes, including diet, weight reduction, and increased physical activity with the goal of reducing total cholesterol to <200 mg/dL and LDL-C <100mg/dL. Various dietary constituents such as green tea, plant sterols, soy protein have important influences on total cholesterol. Medical intervention should be reserved for those patients who have not reached this goal after 3 months of non-pharmacological approach.

**Conclusion:**

CHD remains as a leading cause of death worldwide and hypercholesterolemia is an important cause of CHD. Non-pharmacological methods provide initial as well as long-term measures to address this issue.

## INTRODUCTION

Globally coronary heart disease (CHD) maintains its position as the foremost cause of worldwide deaths. The global burden of CHD is expected to rise. It kills more than 110,000 people in England alone each year with greater than 275,000 people having a heart attack annually. Moreover, over 1.4 million people endure angina every year [1]. The lifetime risk of CHD by 40 years of age was almost half for men (48.6%) and a third for women (31.7%) according to data obtained in the Framingham Heart Study [2]. CHD is the commonest cause of mortality and morbidity globally [3]. Combined with changes in the demographic profile and progressively urbanized lifestyles, westernization in developing countries will result in large numbers of adults who are potentially vulnerable to CHD [4]. Furthermore, there is a linear relationship between cholesterol and CHD mortality [5], the risk of which is reduced upon lowering serum cholesterol levels [6,7].

Therefore, the primary prevention of cardiovascular disease (CVD) by treating asymptomatic hypercholesterolemia represents an important step in reducing coronary morbidity and mortality [8]. While there have been several reviews on cholesterol modifying treatment in the primary prevention of CHD, the evidence of lifestyle modification in cholesterol management is less well documented systematically. The objective of this systematic review is therefore to accumulate a detailed overview of current literature on risk assessment and lifestyle (non-pharmacological) management of incidental asymptomatic hypercholesterolemia.

## RATIONALE FOR MANAGEMENT OF ASYMPTOMATIC HYPERCHOLESTEROLEMIA 

Serum cholesterol level is a major risk factor for CHD. A continuous relationship between low density lipoprotein (LDL) cholesterol and CHD risk is evident and is graded over a wide variety of LDL levels ranging from low to elevated [9]. Higher serum levels of total cholesterol (TC) increased the risk of CHD in the Multiple Risk Factor Intervention Trial (MRFIT) [10]. Even in low serum cholesterol level populations it is clear that lower levels of LDL cholesterol have a direct impact on reduced coronary event rates compared with those patients with relatively elevated serum LDL levels [11]. This is a finding that has been sustained by multiple other studies [5,12,13].

Before considering the impact of lifestyle changes on serum lipids it is first necessary to briefly review the impact of improving lipid profiles on CVD outcomes. A number of pharmacological studies have clearly shown the beneficial results of improved lipid profiles. Although these studies illustrate the results of pharmacological agents and not lifestyle changes, they serve to demonstrate the impact of improving lipid profiles on morbidity and mortality from CVD. These results can be extrapolated for results obtained at set cholesterol levels obtained from lifestyle changes. In hypertensive patients at moderate cardiovascular risk (in the absence of dyslipidemia), a relative risk reduction of 36% in composite primary end points was achieved in the lipid lowering arm of Anglo-Scandinavian Cardiac Outcome Trial (ASCOT-LLA) [14]. There was a reduction of 37% compared with placebo in the incidence of an initial major coronary event, across both sexes upon receiving Lovastatin in the Air Force/ Texas Coronary Atherosclerosis Prevention (AFCAPS/TexCAPS) study [15]. Moreover, on comparing men treated with cholestyramine with men treated with placebo, the former have demonstrated a 19% lower incidence of coronary artery disease according to data obtained in The Lipid Research Clinics Coronary Primary Prevention Trial [8].

There was more than a third reduction (34%) in coronary end point incidence in dyslipidemic men receiving gemfibrozil in the Helsinki Heart Study [16]. A relative risk reduction of almost a third was described in the risk of coronary events with Pravastatin in the West of Scotland Coronary Prevention Study (WOSCOPS) [17]. Diabetic patients with normal or near currently accepted target levels with one other cardiovascular risk factor, have obtained significant reductions in cardiovascular events on taking lipid-lowering 10mg Atorvastatin daily in the Collaborative Atorvastatin Diabetes Study (CARDS) primary prevention trial [18].

To further establish the rationale for managing asymptomatic hypercholesterolemia with lifestyle changes it is worth considering the significant secondary prevention trials published in the literature. The first miletone secondary prevention trial on the topic was the Scandinavian Simvastatin Survival Study (4S) trial. This trial showed a markedly reduced morbidity and mortality associated with CVD in patients using medication to lower cholesterol levels [19]. A reduction in CVD with lower TC and LDL-C levels was further established in the Long-term Intervention with Pravastatin in Ischaemic Disease (LIPID) trial [20].

The Heart Protection Study was the largest of these statin trials and like the 4S trial used Simvastatin to assess outcomes. However, the Heart Protection Study ranged across high risk subjects between both primary and secondary prevention. It demonstrated that all patients using Simvastatin 40mg daily obtained beneficial outcomes no matter their preliminary cholesterol levels [21]. In other words, those patients with lower LDL-C levels obtained similar benefits when compared with patients with elevated LDL-C levels. Higher dose statin, such as Atorvastatin 80mg resulted in a reduction of 22% in primary composite end point and a mean of 2mmol/L LDL-C compared with Atorvastatin 10mg who had a mean of 2.6mmol/L LDL-C. in The Treatment to New Targets trial (TNT) [22].

Statin, Omega 3 fatty acids and dietary modification have been shown to have beneficial outcomes compared to placebo by reducing cardiovascular and overall mortality in lipid intervention studies [23]. There may not be any overt boundary for reduction in LDL-C below which any further cardiovascular benefit cannot be gained. Levels of LDL-C as low as 1.6mmol/L revealed further risk reduction in the Pravastatin or Atorvastatin Evaluation and Infection Therapy-Thrombolysis in Myocardial Infarction (PROVE-IT) study [24]. A range of 1.3 to 1.8 mmol/L for LDL-C is suggested by one study with new research showing even patients in their eighth decade obtain benefit from this [25]. Although these benefits on cardiovascular outcomes are based on pharmacological agents acting to improve lipid profiles, they demonstrate the direct impact that modifying lipid profiles have on prognosis. It is *via* altering lipid profiles that lifestyle changes play such a pivotal role in altering an individual’s risk profile. This article will focus on the important non-pharmacological methods of obtaining similar results on cholesterol levels.

## METHODOLOGY 

A two-step process utilising a Medline/PubMed systematic search was conducted. The initial search was undertaken using elementary phrases including “guidelines for management of hypercholesterolemia”, “non-pharmacological management of hypercholesterolemia”, “risk factors for hypercholesterolemia”, and “management of hypercholesterolemia. Only the most recent literature in the field was required so the time-window for the literature review was restricted to the past 14 years (1998-2012).

The resultant abstracts were analysed and appropriate papers were selected. The secondary search was performed by (1)using the reference lists of the chosen articles and(2) by using PubMedweblinkfor related articles. The studies were selected if they were in English language and included the appropriate topics and if there were in the English language.The search produced over 3600 published papers on the topic of evaluating and managing primary and secondary hypercholesterolemia. All of the reports regarding the evaluation and non-pharmacological treatment of hypercholesterolaemia were chosen.

## LIFESTYLE MODIFICATION OR NON PHARMACOLOGICAL MANAGEMENT OF HYPERCHOLESTEROLEMIA

### Lifestyle Modification 

**Fig. (1):**
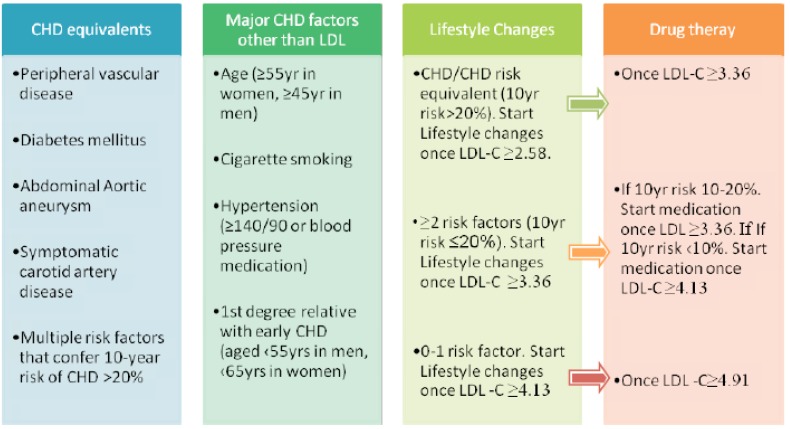
Illustrating the relationship of risk assessment at 10 years against clinical need for intervention at respective LDL-C levels. The figure demonstrates the relation of lifestyle changes to overall management of cardiovascular risk and drug therapy. Risk assessment at 10 years can be calculated using Framingham risk tables or via the risk assessment tool on the ATP III home website. The ATP II guidelines describe how the approach to hypercholesterolemia should be based upon the LDL-cholesterol (LDL-C) fraction in addition to CHD risk factors. CHD equivalents are medical factors which confer the same level of risk as a past medical history of CHD. The presence of CHD, any of the shown CHD equivalents, of multiple risk factors from the second column equating to a 10yr risk of CHD ≥ 20% (as calculated via Framingham risk tables or online ATP III calculation) require early lifestyle changes once LDL-C is ≥ 2.58 mmol/L. In the absence of CHD or CHD equivalents then a higher threshold of LDL-C ≥ 3.36 mmol/L for lifestyle measures can be employed. The final column describes the thresholds at which drug therapy is required in addition to lifestyle factors. All LDL levels are in mmol/L. LDL, Low-density Lipoprotein, CHD, Coronary Heart Disease; LDL-C, LDL-cholesterol fraction. Modified from the ATP III guidelines [28]

**Fig. (2):**
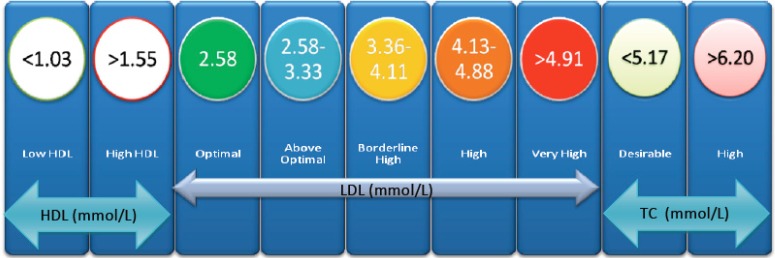
Illustrating the adult lipid profile classification. This diagram allows a clinician to rapidly assess the status of a patient's lipid profile in accordance with the ATP III guidelines. HDL, High-density lipoprotein; LDL, Low-density lipoprotein; TC, Total Cholesterol. Modified from the ATP III guidelines [28]

It is necessary to identify those patients at risk of hypercholesterolemia in order to focus lifestyle modification advice. This is done *via* a CVD risk assessment and it comprises of two main aspects, namely lipoprotein analysis and other risk factor identification. A summary of the risk factors used in the Adult Treatment Panel III (ATP III) guidelines and their relation to lifestyle changes and medical therapy is shown in (Fig. **1**) [26]. The recommended adult lipid profile is summarised in (Fig. **2**).

Once patients at risk of hypercholesterolemia have been identified, their management could be chosen from a range of non-pharmacological options and the exact regimen should be tailored to the individual patient’s lifestyle. Non-pharmacological management of hypercholesterolemia include lifestyle modification such as advising on balanced diet, physical activity, smoking cessation and advice on alcohol consumption, and is the recommended first line of management for patients with total cholesterol of (200 mg/dL and an absolute CVD risk of <20%. These lifestyle interventions are aimed at weight control [27], reducing levels of unhealthy fatty acids such as saturated fat [28] and trans fatty acids (TFA) and improving lipid profile [29-31].

### Diet

In the Seven Country Study, the authors reported that the significant difference at a set cholesterol level in absolute CHD mortality rates may be due to other factors, such as diet, that are typical for cultures with a low CHD risk [5]. Compared with monosaturated and saturated fat, polyunsaturated fatty acids (PUFA) appeared to have a protective effect with regard to arthrosclerosis [32,33]. Numerous studies have looked into the cholesterol lowering effects of dietary intake of plant sterols. However there is no clear consensus on effectiveness and NICE requires further randomised controlled trials before making guidance on this topic [34]. Recently, a federally mandated evidence-based review in the United States concluded that n-3FAs, especially eicosapentaenoic acid (EPA) and docosahexaenoic acid (DHA), have clear cardioprotective effects, and national and international expert panels and health organizations have begun to call for increased EPA and DHA intakes [35].

It has been reported by Keys and colleagues that dietary change can have effect on lipid profile [36]. Dietary modification with a healthy and balanced diet could bring multiple benefits including directly modifying lipid profile [37]. In people with hypertension, dyslipidaemia or diabetes, nutritionally balanced meals that meet the recommendations of national health organizations could improve multiple risk factors for cardiovascular disease including blood pressure, lipids levels, carbohydrate metabolism and weight [38]. ATP III recommends reduced intake of saturated fats to < 7% of total daily caloric intake with reminder of total fats to 25-35% of total energy intake from polyunsaturated and monounsaturated fatty acids [9]. Intake of total cholesterol should be <200 mg/day. European guidelines encourage intake of fruit and vegetable, whole brain cereals, bread, low fat dairy products, oily fish and omega-3 fatty acids [39]. The total fat intake to account for <30% energy intake and saturated fat intake should not exceed a third of total fat intake and total intake of cholesterol should be <300 mg/day [39].

TFA intake has been reported to have adverse effect on LDL, HDL and triglycerides (TG) [33]. TFA are also suggested to be associated with increase in lipoprotein-A and also may increase insulin resistance [40]. Generally, baked foods such as cookies and fried foods are high in TFA and should be avoided. A summary of the recent studies of dietary constituents that may affect the lipid profile and their respective quantitative impact is shown in Table **1**, however a more detailed review of the main ‘functional foods’ that have received attention in the literature are summarised below.

### Fish Oil and Omega-3

In the early 1990’s, it was clear that a fish oil diet had an important impact on serum lipid profile. Harris *et al.* showed that a fish oil diet halved triglyceride levels [41]. This effect is not restricted to hypertriglyceridaemic individuals [42]. Over the years a number of randomised control trials have shown the beneficial action of fish oils in lowering cholesterol [43-46]. A recent meta-analysis of 47 studies demonstrated an average daily intake of 3.25 g of EPA and/or DHA) reduced TG by 0.34 mmol/L (95% CI: -0.41 to -0.27), but did not affect TC, HDL or LDL cholesterol in hyperlipidemic subjects [47].

These results have been translated into clinical outcomes and fish oil dietary supplementation has shown encouraging cardiovascular results. Epidemiologic studies demonstrate reductions in the incidence of nonfatal myocardial infarction and ischemic stroke [48]. There is variation in the effects of omega oils, however an individual’s responsiveness to fish oil could be attributed to his/her ApoE genotype [49]. Small studies have shown fish oils help to slightly lower blood pressure as well as having anti-arrhythmic properties [50-53]. These findings have been incorporated in to national clinical guidelines. NICE guidelines recommend CVD patients consume at least 7 g of omega 3 fatty acids per week from two to four portions of oily fish in the 3 months following myocardial infarction [54]. Numerous studies have reiterated the synergistic action of Omega oils when combined with medical therapy [55-57].

### Soya

Soya contains phytoestrogrens called isoflavones [58]. A recent meta-analysis of 20 parallel-design studies and 23 crossover studies showed a median of 30 g/d of soya protein consumption was associated with a significant improvement in lipoprotein risk factors for CHD [59]. Combining soya foods in conjunction with a prebiotic drinks increased the colonic fermentation and so potentially could increase the hypocholesterolemic effects of soya [60]. Although the isoflavones component of soya does not appear to be the cause of this improvement, it does have other beneficial effects. It has been shown to reduce carotid atherosclerotic burden and improve vascular endothelial function [61].

However, since the chemical structure of isoflavones is similar to oestrogens, it is unclear whether hormonal adverse effects, such as premenopausal breast or gynaecological cancers may be more prevalent with their use [62]. Additionally, although they are advantageous by being low in saturated fats, substituting other proteins for soya has little overall benefit on clinical outcomes and should be considered only as part of a balanced diet [63].

### Plant Sterols and Stanols

Plant sterols and stanols are chemically similar in structure to cholesterol but a methyl or ethyl group in their side chains means they are absorbed weakly in the gastrointestinal tract when contrasted with cholesterol. Plant sterols and stanols are thought to inhibit cholesterol absorption and appear to be active in lowering cholesterol [64]. These compounds are most commonly commercially found in margarines and are a source of their cholesterol lowering marketing campaigns. Table **1** illustrates the plant sterol content of common commercially available margarines [65]. When taken between 2 to 2.5 g/day, products enriched with plant stanol/sterol esters may lower plasma LDL cholesterol levels by 10% to 14% without any reported side effects [66]. A further study has shown daily intake can be increased to 9g, further reducing serum LDL-cholesterol concentrations linearly up to 17.4% [67].

There is no significant difference between plant sterols and stanols in their cholesterol lowering ability [68]. Plant sterols and stanols may have a role in patients with borderline normo/hypercholesterolemia [69]. However there is little evidence directly correlating plant sterol or stanol intake with cardiovascular clinical outcomes. Nonetheless their importance in modifying lipid profiles of patients is reflected in their incorporation in the National Cholesterol Education Program (NCEP) ATP III guidelines on lifestyle changes for elevated LDL-C levels [70].

### Gugalipid 

Gugalipid is derived from the sticky resin of the mukul myrrh tree. It has been used for thousands of years in Indian Aryuvedic medicine to treat a number of ailments. It has received interest in recent years from herbalist and homeopathic professionals with respect to claims it can lower LDL-C levels and slow atherosclerosis. These claims originate from a multicentre clinical trial in India in the late 1980’s showing significant decrease in LDL-cholesterol with the use of Gugga [71]. The active incrediants in gugalipid are the stereoisomers E- and Z-guggulsterone which are antagonist ligands for the bile acid receptor farnesoid X receptor (FXR), which in turn is an important regulator of cholesterol homeostasis [72].

It is *via* this mechanism that gugalipid likely exerts its impact of cholesterol levels [73]. However more recent studies involving gugalipid have failed to replicate these earlier results on lipid profiles with an American double-blind, randomized, placebo-controlled trial actually suggesting it might in fact raise levels of LDL-C [74]. Additionally, skin rash, abdominal discomfort and other adverse outcomes have been described from the use of guggul. A recent paper on the topic concluded that its effects on lipid profiles are still not clear [75].

### Red Yeast Rice

Red yeast rice has been used in Chinese cooking for many centuries. It derives its name from rice fermented by the red yeast, *Monascus purpureus *[76]. The active ingredient is a group of HMG CoA reductase inhibitor-like compounds called monacolins. However other extracts such as Xuezhikang, sterols (discussed above), and monounsaturated fatty acids likely contribute. Red yeast rice has been shown to have significant cholesterol lowering properties [77] however issues over standardized manufacturing practices have been limiting factors to wider promotion [78]. The varying preparations are also an issue when comparing studies of the efficacy of red yeast rice [79].

Xuezhikang may be of benefit in dyslipidemic CHD as it has shown clear lipid-regulating effect on a recent meta-analysis of 22 randomized trials and appears safe and effective in reducing cardiovascular events in these patients [80]. A recent multicentre randomised control trial of 5,000 post-myocardial infarction Chinese patients showed that Xuezhikang decreased CV, total mortality need for coronary revascularization by almost a third each. It also reduced TC, LDL-C, and raised HDL-C was safe and well tolerated [81]. The tolerability of red yeast rice is generally good. A small study has suggested that at doses of 2,400 mg twice daily, red yeast rice shows a comparable reduction in LDL-C when compared with pravastatin (20 mg twice daily) [82].

### Other Cholesterol Altering Dietary Constituents: Green Tea, Garlic, Cocoa and Almonds 

Cocoa products are rich sources of flavonoids, and have been shown to reduce blood pressure and the risk of cardiovascular disease [83]. They can significantly reduce LDL and TC:HDL-C ratio. Several meta-analyses have been conducting showing the beneficial effects of dark chocolate/cocoa products on total and LDL-C [84-88]. Most studies however, have focused on short-term outcomes and longer term data on clinical outcomes is still required [86,87].

The benefits of green tea polyphenols have been widely documented. A recent meta-analysis of 14 studies demonstrated that green tea resulted in significant reductions in serum TC and LDL-C (by 0.06mmol/l), but with no effect on HDL-C. Two recent large prospective studies; the 2006 Ohsaki study [89], and the 2009 Shizuoka elderly cohort [90] both noted reduced all cause and CVD-related mortality due to and due to cardiovascular disease with Green tea consumption.

A small number of meta-analysises have assessed the literature on the effects of garlic on lipid profiles [91-94]. Garlic may modestly reduce TC levels, but has no real impact on LDL lowering or HDL elevation however these meta-analyses are limited by the methodological quality of existing papers on the topic [91,93]. Limited evidence has suggested 25 to 168 g/day of almonds may significantly lowered total cholesterol [95] but insufficient to promote almond ingestion as part of a lipid-lowering regime. Virgin olive oil and a Mediterranean diet have well documented lipid lowering properties [96-98]. The quantitative evidence for these is summarised in Table **1**.

### Physical Activity

Physical inactivity is a major underlying risk factor for CVD. It is also independent of other risk factors such as obesity and hypertension [99]. Physical activity or physical fitness have been shown to be associated with reduction in the risk of CVD [100]. Regular physical activity reduces VLDL levels, raises the HDL cholesterol, and to a lesser extent, lowers the LDL levels [101]. Other benefits of regular physical activity include lowering of blood pressure [102,103] and reduction in insulin resistance [104]. The recommendations vary but the general consensus view is that at least 30 minutes of moderate intensity physical activity should be encouraged three to four times a week [105].

Studies from the 1970’s showed a dramatic disparity between the lipid profiles of active and sedentary men [106]. Unsurprisingly exercise regimes have greatest impact on an individual’s lipid profile when combined with dietary changes [107]. The general concensus of contemporary research in the field is that amount of exercise plays a far greater impact on serum lipid profiles than the intensity of exercise [108,109]. There is a clearly graded improvement of lipid profile with amount of exercise [110]. The type of exercise has been the focus of some debate, however generally both aerobic exercise and progressive resistance training improve lipid profiles in adults [111-113].

There are significant gender differences on the effect of exercise on cholesterol levels. HDL-C levels increased significantly more in the men who exercised and dieted than in women [114]. The cause of these differences and the underlying mechanism by which exercise exerts its effect on cholesterol levels is incompletely understood. Exercise reduces hepatic lipase activity and increases lipoprotein lipase activity [115]. Peak LDL particle diameter and HDL2-mass increases with exercise and correlates with the changes in lipid composition [116,117]. These changes are equally important across all age ranges [118,119].

### Weight Control

Obesity is also recognized as an independent major risk factor for CHD [120]. Weight reduction therapy for overweight or obese patients, will enhance LDL lowering and provide other health benefits including modifying other lipid and non-lipid risk factors [121]. A large study from Finland suggests cardiovascular outcomes in obese children who subsequently lost weight by adulthood and became non-obese were analogous with those who were never obese [107]. Weight loss is currently recommended for individuals with a body mass index (BMI) >=30 kg/m^2^ (obese) and in individuals with a BMI 25-29.9 kg/m^2^ (overweight) when 2 or more risk factors are present. Waist circumference of >102 cm in men and >88 cm in women is also a risk factor for CHD. A specialist dietician should participate actively in the dietary management of these patients.

### Smoking

Smoking cessation is possibly the single most significant lifestyle change an individual can make as it is a major modifiable risk factor for CVD [122-125]. It has a synergistic adverse effect in combination with high cholesterol levels. Nicotine replacement therapy is proven as adjunctive therapy to increase the probability of quitting smoking [126]. Buproprion (Zyban) is a selective reuptake inhibitor of Dopamine and Noradrenaline which prevents or reduces cravings and other features of nicotine withdrawal. Buprenorphine SR is useful oral non-nicotine therapy for smoking cessation [127]. Individual counselling from a smoking cessation specialist is also better than no counselling [128], as are group programmes [129].

Smoking increases TC, and lowers HDL-C and raises LDL-C [130,131]. Initial low cholesterol levels prior to initiating smoking confer no protective benefit against smoking-related atherosclerotic cardiovascular disease [132]. The adverse outcomes on LDL-C and HDL-C are worse in younger smokers between 8- to 19-years-old [131]. Cigarette smoking cessation increases serum levels of HDL-C but not of TC, LDL-C, and TG [133] .

### Alcohol Consumption

The link between alcohol and lipid profiles has been well established for several decades. In the 1960s, hyperlipidaemia across all lipoprotein classes was noted in rats after feeding them with alcohol [134-136]. Since then it has been extensively noted that alcohol significantly increases levels of high density lipoprotein cholesterol and results in favourable changes in several cardiovascular biomarkers in human studies [137,138]

Epidemiological studies are consistent in showing that light to moderate alcohol intake has an inverse association with the risk of cardiovascular disease morbidity and mortality compared with those who do not drink or who consume alcohol in excess amount [139]. Alcohol is thought to reduce the risk of cardiovascular disease through increases in HDL cholesterol and is likely to be of more benefit in combination with a healthier lifestyle such as remaining physically active [140]. The current recommendation set forth by the American Heart Association and other groups is to limit alcohol intake to no more than 2 drinks per day for men and 1 drink per day for women.

Drug therapy should be considered for those patients who have failed to attain the goals of lowering the LDL-C<100mg/dL and reducing total cholesterol to <200 mg/dL after 3 months of the above detailed non-pharmaceutical approaches.

## FACTORS AFFECTING COMPLIANCE WITH LIFESTYLE CHANGES

Despite the extensive literature on the benefits of the above lifestyle changes on lipid profile, there are a number of factors affecting patient compliance with lifestyle advice. Factors contributing to poor compliance after counselling on lifestyle changes are summarised in Table **3**. There have been a number of theories to conceptualise the process of lifestyle modification. In relation to lifestyle modification for the treatment of hypercholesterolemia, two main theories are significant and largely overlapping; the transtheoretical model of health behaviour change [141] and the health belief model [142]. Their relationship is summarised in Table **4** [141,142].

Factors affecting cognitive ability such as dementia, brain injury or level of education will directly impact comprehension of the significance of lifestyle changes. Reversible factors affecting cognition such as clinical depression act as barriers in effective lifestyle stage [143]. Poor patient motivation acts as a barrier to the precontemplative stage of potential lifestyle modification and can prove difficult hurdle to pass. The best approach appears to be clear patient communication in a professional healthcare setting [144]. The use of motivational interviewing techniques appears to enhance weight loss in overweight and obese patients [145,146].

Complex dietary advice and intensive change is likely to fail. Adverse outcomes of exercise regimes such as tiredness, musculoskeletal issues, or social embarrassment should be addressed with empathy to improve patients' weight-related attitudes [147]. Patients should be regularly followed up to assess their progress as well as to address any of their queries [148]. Frequent telephone contact with a dietician was found to be as effective as face-to-face dietician contact for supporting lifestyle modification in obese patients trying to lose weight [149].

## DISCUSSION

LDL-C alone is not sufficient to accurately represent the complete atherogenic lipid profile. Evidence from studies including the PROCAM [13] and Strong Heart Study [150]propose that HDL-C levels are in fact the major lipid predictors of CVD risk. However, lowering cholesterol continues to be key to the outcomes of statin treatment on CVD prevention according to the results of ALLHAT-LLA trial [151], whereas the outcome benefits of cholesterol reduction are augmented by greater HDL-C levels.

Even though the underlying risk factors regarding the progression of CHD are identical in both sexes, there are a number of quantitative differences. However, as yet there has not been any formal clinical trial focusing specifically on cholesterol lowering in women. Nonetheless, cholesterol lowering is as beneficial in women as in men according to a recent meta-analysis of new statin trials with clinical end points. The major controversy in cholesterol screening is whether to screen young adults. Guidelines exclude many persons 65 years and older as well as men and women younger than 35 and 45 years respectively. They also do not recommend measurement of HDL-C.

Even though it has been widely accepted that atherosclerosis is a process which begins at an early stage, there has not been any scientific data to base clinical decisions on how young a patient should be before treatment is considered. The crux of the matter is whether patients under 20 years old should be routinely tested? Additionally, should treatment be started even earlier for high risk people? Unfortunately these are the questions which still remain unanswered. Further controversy arises on the need for aggressive management of hyperlipidaemia in an elderly population over 65 years of age. The debate arises on the fact that cardiovascular morbidity and mortality in this age group is well recognised increase with ageing yet there has not been much formal evidence to suggest that lipid reduction in this age group has positive effect on outcomes. However, using the data from the Scandina*via*n Simvastatin Survival Study (4S) in form of sub-analysis has demonstrated that lowering LDL-C in this elderly subgroup reduced all-cause mortality risk by over a third, which equates to a CHD risk-reduction of 43% [19].

A similar 32% relative risk reduction in patients aged 65-75years of age was elicited in a sub-analysis of Cholesterol and Recurrent Events Trial (CARE) [152]. Although both the CARE and the 4S studies tackled risk reduction secondary prevention, there is good evidence for its role in primary prevention. The AFCAPS/TexCAPS study was a primary prevention trial and subgroup analysis of this trial described a reduction in the rate of CHD events to those similar in young individuals in women > 62years of age and men >57 years of age. The Prospective Study of Pravastatin in the Elderly (PROSPER) study has demonstrated a relative reduction of 15% in the risk of primary end points by treating elderly patients (70-82 years old ) with Pravastatin [153].

Patient who are informed as being at ‘high-risk’ are more enthused to make lifestyle changes and will reap more substantial individual benefits from reducing risk factors. This is the advantage of a target high-risk population epidemiological approach to hypercholesterolemia. It provides a cost-effective means to target those who will proportionally suffer greatest CHD morbidity and mortality.

However, it is clear that many more people will suffer strokes and coronary events with near average cholesterol levels by sole virtue of the generically elevated risk within the populationin its entirety. As a result, this ‘high risk’ approach cannot target the vast majority of the population who will actually suffer from the disease. A further example is the clear association between hypertension and cerebrovascular disease, however many patients who have suffered a stroke have not had hypertension. It is simply due to the numerically greater numbers of people will suffer from greater numbers of strokes (even though proportionally may be a lower risk than hypertensive patients). If the general population cholesterol levels were not so high then the issue of drug based intervention to large numbers of the high risk population would not be such an issue. A recent Dutch study suggested over 50% of patients in primary care did not have sufficient indication for medication based on the Dutch cardiovascular risk management guidelines.

A different approach to the prevention of CHD aims to control the determinants of population incidence as a whole [154]. This is termed the ‘population strategy’. The risk associated with many clinical variables [155] is continuous with no evidence of threshold. Such variables are strong, consistent predictors of cardiovascular disease in groups, but are poor predictors of susceptibility in individuals. Plotting the population distributions of five groups with increasing median values of risk reveals that their normal distributions shift rightward. Although a study based on the recent Dutch guideline for cardiovascular risk management suggests over half of primary care patients may have insufficient indication for medication [156], a reduction of 10% in cholesterol levels in the entire population has been predicted to reduce coronary heart disease mortality by 20% [157].

Additionally, Framingham data suggests that a 10 mm Hg lowering of the blood pressure distribution as a whole would correspond to about a 30% reduction in the total attributable mortality [154]. To create an environment in which individual behavioural initiatives can succeed, major shifts in population behaviour through public health policy are necessary. Population-level health promotion through government or non-governmental organisations such as disease-specific charities aims to increase awareness of good health and improve access to it. The North Karelia project in Finland began in 1972 at a time when the country had one of the highest rates of cardiovascular disease in the world. The project targeted principally dietary behaviour to reduce cholesterol levels and led to a decrease in cardiovascular incidence in the region [158]. The project influenced dietary behaviour throughout the country with time, and also led to changes in the food industry with increased production of healthier cooking oils and salt reduction in food products. Thus, tackling a country’s unhealthy diet and unhealthy food production policies should be part of the therapy.

The great variability in absolute mortality rates from coronary heart disease at certain cholesterol levels suggests that different factors, both dietary and non-dietary would possibly contribute to this idea. An example supporting this idea in the South Asian population in the UK who have paradoxically high rates of coronary disease despite relatively low total cholesterol and implies other factors may be at work [159]. It is by virtue of this that a global risk stratification should be conducted incorporating all factors and not just high cholesterol as a single entity. An example of this would be the fact that the initiation of medical treatment will be of little help if the person continues smoking and if blood pressure remains poorly controlled [160]. Statistics recently obtained from Britain between 1998 and 2003 demonstrate the reduction in cholesterol has leveled off as obesity has gradually increased [161].

Hence addressing these non-pharmcological factors globally and especially amongst developing countries with progressively urbanized lifestyles are major priorities. This article has described the components of lifestyle modification towards addressing hypercholesterolemia without pharmacological intervention. The evidence of these therapies are growing, and there is already strong support for the impact of green tea catechins, Plant sterols.stanols cocoa, soy protein isoflavones and a Mediterranean diet in the managementof hypercholesterolemia (Table **1**). There is little evidence on whether the beneficial effects on lipid profiles may be multiplied on combining these dietary items. However, a trial of 3 months for lifestyle modification prior to considering pharmacological intervention is warranted by a number of cardiovascular guidelines [162,163].

## CONCLUSION

CHD is a leading cause of death in industrialised nations. Hyperlipidaemia with elevated serum LDL-C, total cholesterol and triglycerides is a known major cardiovascular risk factor. HDL-C is protective and low HDL-C is recognized as an independent cardiovascular risk factor. Detection and treatment of elevated cholesterol levels in young adults has the potential to prevent premature CHD as well as CVD. The new Joint British Guidelines recommend that clinicians pay equal attention to apparently healthy individuals who are at risk of going on to develop CVD. Based on data from HPS and CARDS trials it may be sensible to start statin treatment on the basis of global cardiovascular risk assessment instead of simple pre-treatment cholesterol values.

In summary, patients with total cholesterol of (200 mg/dL and an absolute CVD risk of <20% should be advised about lifestyle changes, including diet, weight reduction, and increased physical activity with the goal of lowering the LDL-C<100mg/dL and reducing total cholesterol to <200 mg/dL. Drug therapy should be considered for patients who have failed to reach this goal after 3 months of non-pharmacological approach.

Intensive drug treatment should be initiated if there are a number of risk factors bestowing 10-year CVD risk of greater than 20%. Drug therapy should be considered in those patients with LDL-C of130 mg/dL or more [160]. Patients who warrant intensified therapeutic lifestyle changes and drug treatment include those with diabetes mellitus, dyslipidaemia, hypertension, family history of premature CHD, or a combination of these risk factors, since these patients are at high risk of developing CVD [160]. A patient’s absolute risk ofcardiovascular eventsis more significant than the simple pre-treatment lipid values. The objective of therapy should be to achieve considerable absolute reductions in LDL-C and total for that patient. Patients should be monitored vigilantly for compliance and any adverse side effects.

### Take-home Message for the Clinicians

CHD remains as a leading cause of death worldwide and hypercholesterolemia is an importance cause of CHD.

Asymptomatic hypercholesterolemia in the presence of other risk factors for CHD may be associated with significant risk for CHD morbidity and mortality.

Currently recommended guidelines suggest “the lower the better” approach with regard to LDL-C and total cholesterol.

Target cholesterol levels set out in various guidelines could be achieved by lifestyle changes, including diet, weight reduction, and increased physical activity with the goal of lowering the LDL-C<100mg/dL and reducing total cholesterol to <200mg/dL. Drug therapy should be considered for patients who have failed to reach this goal after 3 months of non-pharmacological approach.

## Figures and Tables

**Table 1.** The Influence of Dietary Elements to Total Cholesterol

Change in Cholesterol	Red Yeast Rice^1^	Guggulipid^2^	Almond consumption^3^	Garlic^4^	Green tea catechins^5^	Chitosan^6^	Plant Sterols/ Stanols^7^	Virgin Olive Oil^8^	Meditaranian diet^9^	Cocoa^10^	soy protein	isoflavones^11^
LDL change (mmol/L)	↑1.11-1.19	↓0.17	↑0.15 mmol/L	Unaffected	↑0.14	Unaffected	↑0.35	↑0.08	↑0.09**	↑0.15	↑0.21
HDL (mmol/L)	Unaffected	Unaffected	Unaffected	Unaffected	Unaffected	Unaffected	↑0.36	↓0.045	↓0.03	Unaffected	↓0.04
TC(mmol/L)	1.04	Unaffected	↑0.18 mmol/L	Unaffected	↑0.14	↑0.30	↑0.1	↑[phenol]*	↑0.19	↑0.15+	↑0.22
Level of Evidence	B	C	B	B	A	A	A	B	A	A	A

1 =41 patients were given capsules containing either rice powder placebo vs. 42 patients given 2.4 g red yeast rice daily with serum measurements at 8 and 12 weeks [75]. A further randomized trial involved giving 1800 mg to 31 patients with 12-24week follow up [161].

2 =Data based on a double-blind, randomized, placebo-controlled trial using a parallel design [73]. However conflicting data available from older studies [70, [71]

3 =25 to 168 g/day significantly lowered total cholesterol [92]. However, insufficient body of evidence to promote almond ingestion as lipid-lowering regime.

4 =Data from parallel-design randomized clinical trial involving 192 adults [162].

5 =Data from meta-analysis from twenty trials GTCs at doses ranging from 145 to 3,000 mg/day taken for 3 to 24 weeks (N=1,415) [163].

6 =Data from meta-analysis from Six randomized, placebo-controlled trials of chitosan in hypercholesterolemic patients (n = 416 patients) [164].

7 =Data from a systematic review with meta-analysis of 20 studies showing foods enriched with 2.0 g of phytosterols/stanols per day had a significant cholesterol lowering effect [165]. There is no significant difference between plant sterols and stanols in their cholesterol lowering ability [166].

8 =Data based on a multicentre randomized, crossover, controlled trial conducted at 6 research centers from 5 European countries. 200 healthy male participants were randomly assigned to 3 sequences of daily administration of 25 mL of 3 olive oils of varying phenolic content at 3 weeks intervals preceded by 2-week washout periods [93]

* decrease in TC linearly dependent on phenol concentration of olive oil consumed.

9 = Data obtained from Meta-analysis which identified 6 trials, including 2650 individuals [94]. A further meta-analysis included 50 original research studies (35 clinical trials, 2 prospective and 13 cross-sectional), with 534,906 participants [95].

** (widely quoted but statistically not significant).

10 = data obtained from meta-analysis involving eight trials (215 participants). (+= total cholesterol lowered by 0.15(mmol/L)), however statistically insignificant at p=0.08 [83].

11 =Data obtained from meta-analysis involving twenty-three eligible randomized controlled trials published from 1995 to 2002 [58]. Level of Evidence: A= Systematic Review, B= Randomised control Trials, C=A large degree of conflicting data between studies.

**Table 2.** Illustrating Common Commercially Available Margarines which Contain Plant Sterols

	Flora Pro-activ^™^Original^™^	Promise Activ^™^ spread	BENECOL^®^ Light Spread
	1 tablespoon	2 tablespoons	2 tablespoons
Directed daily intake (amount of Plant Sterols)	2g	2.240g	1g
Daily recommended amount of Plant Sterols	1 to 3 grams of plant sterols per day lowers LDL cholesterol by 5-15% [65]

**Table 3.** Factors Contributing to Poor Compliance After Counselling on Lifestyle Changes

Factor	Impact on Compliance	Impact on Success Rate
	*Patient Factors*	
Poor Patient Motivation	↓	↓
Poor Cognition	↓	↓
Inaccurate Health beliefs (poorly perceived benefits of change)	↓	↓
	*Clinical factors*	
Lack of Clinical follow up	↓	↓
Adverse outcomes of regime^1^	↓	↓
Complexity of regime^1^	↓	↓

*Note:* ^1^Regime is defined as any combination of dietary or lifestyle change

**Table 4.** The Process of Lifestyle Modification for Hypercholesterolaemia Divided into Key Components

Patient’s Stages of Change^1^	Health Behaviour Model^2^
1) Precontemplation	1) Perceived threat
2) Contemplation	
3) Preparation	2) Outcome expectations
4) Action	
5) Maintenance	3) Efficacy expectations

1 Based on the transtheoretical model of health behaviour change [139].

2 Based on the health belief model [140]
